# Sediment Microbiota in Response to Circuitry of Sediment Microbial Fuel Cells, Revealed by 16S rRNA Gene Amplicon Sequencing

**DOI:** 10.1128/MRA.00984-21

**Published:** 2021-11-18

**Authors:** Yasuyuki Takemura, Masataka Aoki, Thao Tran P., Noriko Tomioka, Keiichi Kubota, Norihisa Matsuura, Kazuaki Syutsubo

**Affiliations:** a Regional Environment Conservation Division, National Institute for Environmental Studies, Ibaraki, Japan; b Department of Civil and Environmental Engineering, Nagaoka University of Technology, Niigata, Japan; c Division of Environmental Engineering Science, Faculty of Science and Technology, Gunma University, Gunma, Japan; d Faculty of Geosciences and Civil Engineering, Kanazawa University, Ishikawa, Japan; University of Southern California

## Abstract

Information about sediment microbiota affected by sediment microbial fuel cells (SMFC) is limited. A laboratory-scale SMFC was applied to a eutrophic lake sediment under closed-circuit/open-circuit conditions. We analyzed the prokaryotes in the sediment adhering to the anode material. The archaeal family *Methanoperedenaceae* was a predominant group under closed-circuit conditions.

## ANNOUNCEMENT

Sediment microbial fuel cells (SMFC) can generate electrical energy using an anode embedded in the sediment and a cathode submerged in the overlying water (1). In this system, exoelectrogens can transport extracellular electrons to the anode, and the electrons are transferred to the cathode via the circuit. The SMFC has recently attracted attention as a renewable energy source and a promising environmental remediation technology (2). However, information on exoelectrogenic microbiota in eutrophic freshwater lake sediments is limited. Here, we provide 16S rRNA gene amplicon profiles of the microbiota attached to the anode materials under closed-circuit (CC) and open-circuit (OC) conditions.

The SMFC column configurations have been described previously (3). Briefly, the surface sediment (depth, 0 to 20 cm) and surface water samples were collected using an Ekman grab sampler from Lake Kasumigaura, a eutrophic freshwater lake in Japan. The SMFC columns (sediment thickness, 12 cm; water depth, 17 cm) were constructed in a plastic cylinder (diameter, 6.3 cm). Carbon felt (Torayca mat BO050; Toray Industries, Japan) was used as the electrode material (anode depth, 5 cm; cathode depth, 10 cm) with an external resistor (1 kΩ) under CC conditions. Four types of columns were prepared in duplicate for different sediment and circuit conditions. Columns A and C used unspiked sediment, whereas columns B and D used sediment spiked with sodium acetate (final concentration, 10 mM). The column operations were initiated under OC (columns A and B) and CC (columns C and D) conditions. On day 64, the circuit conditions were switched from OC to CC (columns A and B) or from CC to OC (columns C and D). SMFC electricity generation was successfully demonstrated under CC conditions for all of the columns (3). On day 224 (160 days after the switching), 0.5-g (wet weight) samples of the sediment adhering to the anode materials in the eight columns were scraped and collected (Table 1). The sediment samples were freeze-dried using an VD-250R lyophilizer (Taitec, Japan) and were subsequently subjected to DNA extraction using a GenCheck DNA extraction kit (FASMAC, Japan) (4). The amplicon libraries of the V4 region of prokaryotic 16S rRNA genes were prepared by a two-step tailed PCR method (5) with 515F and 806R primers (6). The resulting libraries were sequenced at FASMAC using the MiSeq platform and MiSeq v2 reagent kit (Illumina, USA). Microbiome analysis was carried out using QIIME 2 v2020.08 (7). The 2 × 250-bp paired-end reads were trimmed (options: TRIMLF = 20, TRIMLR = 21, TRUNCLENF = 230, and TRUNCLENR = 210), and the amplicon sequence variants (ASVs) were generated using DADA2 implemented in QIIME 2 (8). The taxonomic classification of each ASV was performed using the SILVA 138 database (9).

**TABLE 1:** Profiles of sediment samples and 16S rRNA gene amplicons

Column type and sample no.	Sediment type^*a*^	Electrical circuit conditions^*b*^	No. of raw sequencing reads	No. of ASVs	Good’s coverage index	SRA accession no.
A (sample 1)	sed	CC	127,474	100,140	0.977	SRR16131133
A (sample 2)	sed	CC	130,799	102,669	0.977	SRR16131132
B (sample 3)	ace+	CC	115,947	92,144	0.978	SRR16131131
B (sample 4)	ace+	CC	127,486	100,600	0.978	SRR16131130
C (sample 5)	sed	OC	137,319	108,563	0.978	SRR16131129
C (sample 6)	sed	OC	110,506	86,912	0.975	SRR16131128
D (sample 7)	ace+	OC	138,557	108,637	0.978	SRR16131127
D (sample 8)	ace+	OC	131,619	103,025	0.975	SRR16131126

a sed, Lake Kasumigaura sediment without acetate spiking; ace+, sediment spiked with acetate.

b Electrical circuit conditions were noted when the samples were taken.

**FIG 1:**
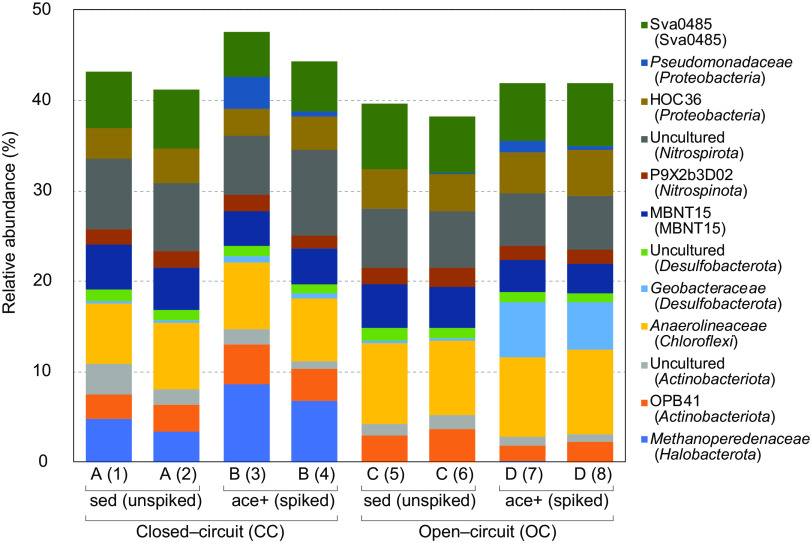
Relative abundance of prokaryotic communities at the family level (more than 2% in at least one of the analyzed samples) in the sediment samples adhering to the anode materials. The names in parentheses denote the phylum-level classifications.

The archaeal family *Methanoperedenaceae* was the only group that showed a significant difference with the circuit condition (Fig. 1). *Methanoperedenaceae* was predominant under CC conditions (columns A and B; relative abundance, 5.89% on average), compared to OC conditions (columns C and D; relative abundance, 0.03% on average) (*P *< 0.005 by Student's *t* test). These data imply that this archaeal group may contribute to the bioelectrogenesis of the SMFC in eutrophic lake sediments.

### Data availability.

The sequences were submitted to the NCBI Sequence Read Archive (SRA) under the BioProject accession number PRJNA767541 (Table 1).
